# Does adopting a healthy diet improve periodontal parameters in patients susceptible to periodontal disease? A systematic review

**DOI:** 10.1038/s41432-024-01098-0

**Published:** 2025-01-10

**Authors:** Charlotte Glavin, James Gartshore, Guy Jackson, Steve Bonsor

**Affiliations:** Edinburgh University, Edinburgh, UK

**Keywords:** Gum disease, Nutrition and diet in dentistry

## Abstract

**Objectives:**

The aim of this systematic review is to evaluate evidence relating to whether adopting a diet, associated with improved outcomes for chronic systemic diseases with an inflammatory component, can improve periodontal parameters in patients with periodontal diseases.

**Data sources:**

Electronic databases and one platform were systematically searched; Medline, Embase, Web of Science and the Cochrane Library including references of relevant studies.

**Data selection and extraction:**

1220 studies were identified of which 9 studies were eligible; 4 RCT’s, 1 controlled trial and 4 observational cohort studies

**Data synthesis:**

8 out of 9 studies found improved periodontal parameters associated with a diet that was low in refined carbohydrates, low in saturated fats, high in fibre and high in nutrition but the studies were low to medium quality of evidence and the diets, method of recording the diets and periodontal parameters varied between the studies as did the study duration and age of participants.

**Conclusion:**

Current evidence supports the hypothesis that adopting a healthy diet has the potential to positively impact periodontal parameters in patients with periodontal diseases, particularly in the older population but the effects may be negated by confounding factors such as smoking. 9 studies were included in the review which were rated moderate or low quality of evidence.

Key points

- Informs the public that adopting a healthy lifestyle and diet may improve gum health.
- Informs the public that smoking significantly lessens improvements in gum health associated with adopting a healthier diet.
- Informs health care professionals that taking a holistic approach to treatment planning, by including discussion of the benefits of healthy eating habits, may improve overall outcomes for patients with periodontal diseases.
- Informs the public of the possible associations between systemic inflammation, periodontal disease and diet.

## Introduction

Chronic inflammation of the soft tissues associated with the dentition is termed, ‘gingivitis’. Destruction of the periodontium and supporting alveolar bone, attributable to chronic inflammation, is referred to as, ‘periodontitis’. Currently an estimated 90% of the worldwide population is affected by gingivitis and around 11% is affected by severe periodontitis^1–3^. Gingivitis and periodontitis are commonly referenced under the umbrella term of, ‘periodontal diseases’.

Susceptibility to periodontal disease arises when pathogenic oral microbes, such as Porphyromonas gingivalis, Aggregatibacter actinomycetemcomitans and Tannerella forsythia, aggregate in sufficient numbers adjacent to the dentition to initiate a host immune response. The global prevalence of periodontal disease should be a cause for concern as the pathogenic periodontal microbes may play a role in exacerbating systemic chronic inflammatory diseases such as atherosclerosis, diabetes, arthritis, non-alcoholic fatty acid liver disease (NAFLD), Alzheimer’s Disease, kidney disease and inflammatory bowel diseases^4–11^.

Tooth brushing with interproximal cleaning, is the established method for reducing periodontal diseases via removal of pathogenic organisms from the surfaces of the host’s dentition^12–14^, However, health and lifestyle factors can also influence the severity of periodontal disease progression. It has been established that tobacco smoking can negatively affect periodontal health but other factors have also been associated with changes in periodontal health such as arthritis, nutritional imbalance, pregnancy, stress, sleep disturbance and frequency of exercise^15–26^.

Heart disease, diabetes and obesity are medical conditions which have an inflammatory component and are also known to have an association with the severity of periodontal disease^27^. An, ‘unhealthy diet’, alternatively referred to as, ‘a western diet’, consists of foods that are high in refined carbohydrates, high in saturated fats, low in fibre and low in nutritional value and these diets are associated with diminished outcomes for heart disease, diabetes and obesity, often in conjunction with other unfavourable lifestyle factors such as increased frequency of smoking or lack of exercise^16,28–31^.

A ‘Healthy’ diet, alternatively referred to as, ‘Mediterranean’, ‘Optimised’, Okinawan’, or ‘Low Inflammatory’, consists of foods that are low in refined carbohydrates, low in saturated fats, high in fibre and high in nutritional value and is associated with improved outcomes for heart disease, diabetes and obesity, often in conjunction with favourable lifestyle factors, like increased frequency of exercise^30,32–44^. Foods commonly associated with healthy diets include nuts, berries, fruit, vegetables, wholegrain foods, whole foods, healthy oils and fish^30,43,45,46^.

The association between systemic chronic inflammatory diseases and periodontal disease, informs the question: Does adopting a diet associated with improved outcomes for chronic systemic diseases with an inflammatory component, such as heart disease, obesity or diabetes, also improve periodontal parameters in patients with gingivitis or periodontitis?

The aim of this systematic review is to establish whether a diet associated with improving general health contributes to a reduction in periodontal disease by systematically searching and assessing the current available evidence to inform recommendations for targeted, individualised dietary advice that could be incorporated into the management and prevention of periodontal disease.

## Methods and materials

PRISMA, Preferred Reporting Items for Systematic Reviews and Met-Analysis, principles were applied when designing and implementing the question and research strategy for the systematic review using the PRISMA checklist.

The principles of PEO, Population, Exposure and Outcomes, were applied and presented in Table 1.

**Table 1:** Table of PEO objectives.

Initial	Objective
Population	Adult global population, 18 and over
Exposure	Maintaining or adopting a diet associated with improved general health
Outcomes/measures of effect	Improved periodontal markers with time.
Studies	RCT’s, case-control studies, cross-sectional studies, population survey studies

### Protocol and registration

The systematic review was registered with PROSPERO, ID CRD42022359374, (12^th^ September,2022), https://www.crd.york.ac.uk/prospero/display_record.php?ID=CRD42022359374.

### Source and search strategy for electronic databases

The search strategy included an electronic search of the databases, Medline, Embase and the Cochrane Library. An electronic search of the platform Web of Science. Manual searching of references from all eligible studies and forward citation searches of manuals related to the topic. Publishers of the Journal of Clinical of Periodontology and The Journal of Periodontology, along with authors of studies related to the PICO question were contacted by email to enquire if they were aware of any related unpublished studies or ongoing studies.

### Study and data extraction

#### Eligibility criteria

Table 2, lists the inclusion criteria and Table 3 lists the exclusion criteria applied to the studies identified from the search strategy.

**Table 2:** Table of inclusion criteria.

1	RCT’s, case-control studies, cross-sectional studies, population surveys
2	Measurement of periodontal parameters by a qualified examiner on the participant (BOP, C.AL, PPD, GI)
3	Comparison of periodontal parameters between diets associated with optimising general health and diets associated with negatively impacting general health
4	Measurement of change with time, minimum 4 weeks
5	Any country
6	Human only
7	In vivo studies
8	20 or more participants
9	All languages where translation is possible
10	18 and over

**Table 3:** Table of exclusion criteria.

1	Animal trials
2	Less than 20 participants
3	No measurement of periodontal parameters, parameters only taken from radiographs, self-reported periodontal parameters.
4	No comparison of periodontal parameters between diets associated with optimising general health and diets associated with negatively impacting general health
5	Individual foods, i.e., single foods rather than food groups
6	Beverages
7	Food supplements in the absence of whole foods
8	Individual case reports
9	Studies using the same data sources
10	Elimination diets including vegetarian and vegan diets
11	Expert opinion
12	Unretrievable data

#### Diet analysis

The DII, (Dietary inflammatory index), HEI, (Healthy Eating Index), MDS, (Mediterranean Diet Score), MEDAS, (Mediterranean Diet Adherence Screener), DASH, (Dietary Approaches to Stop Hypertensions), BSDS, (Baltic Sea Diet Score), RFDS, (Recommended Finnish Diet Score) and the HFD-index, (Healthy Food Index) are tools designed to assess and quantify the quality of diet with regards to the impact it has on general health^34,36,38^. Although the scoring systems differ, there is consensus on which food groups are considered healthy and which are considered unhealthy^31,43,44,47,48^.

#### Age

Participants under 18 years old were excluded because detection, presentation and progression of periodontal diseases in children and young adolescents can differ when compared with adults^49,50^.

#### Participant numbers

Evidence varies on the recommended minimum number of participants for RCT’s in medical studies, some recommend no less than 30 participants whilst others prefer a minimum of 100 participants^51,52^. During preliminary searching it became apparent that high quality randomised control trials in this research category had limited numbers of participants, which varied from as low as ten participants to above 60. Dietary compliance combined with eligibility and financial restrictions may be the reason for the low numbers^53^. A minimum of 20 participants or studies that demonstrated a power calculation was applied to the inclusion criteria to reduce the impact that studies with very small sample sizes could have on the overall results^54^.

#### Exclusion of elimination diets

Elimination diets were excluded from the inclusion criteria, including keto diets, vegetarian and vegan diets due to increased associations with nutritional imbalance, which could negatively influence the periodontal parameters within the studies^55–57^.

#### Exclusion of beverages and supplement studies

Studies solely reviewing supplements or beverages were excluded as they were unlikely to contain dietary fibre which may contribute to general health via maintaining a healthy biome and aid nutrient uptake^46,58–61^.

#### Single food items

Studies of one food item, were excluded, as it is impractical to implement consumption of specific foods on a global scale due to availability issues, interactions with medications, food sensitivities, allergies, economic affordability or religious and cultural practices^62,63^.

#### Data extraction

The search strategy and search results for each of the electronic databases Medline, Embase, Cochrane Library and Web of Science are available in supplementary data as Supplementary Appendix A, B, C and D respectively. The date the searches were performed was the 20^th^ of November, 2022. Titles and abstracts obtained from the electronic searches were screened, using the inclusion/exclusion criteria. The titles and abstracts of eligible papers were searched, by two independent assessors and papers that did not meet the criteria excluded from the review. The remaining complete papers were then studied and included or eliminated using the eligibility criteria. Disagreements regarding eligibility were settled using a third independent examiner. A proforma for abstracts and full papers was provided to reduce the risk of selection bias with reasons for exclusion of studies systematically recorded in the selection tool provided, (Supplementary data, Supplementary Appendix E).

Duplicate papers or data were eliminated. Further searches were performed via references of relevant studies and contacting relevant authors. All languages were included, however if translation into English was unavailable the paper was excluded.

#### Risk of bias in individual studies

Selected study outcomes were assessed for risk of bias using the Cochrane risk of bias tool, (RoB 2), for randomised trials and ROBINS-I tool for non-randomised trials and ROBINS-E tool for any cohort or case control studies. The risk of bias across the trials was assessed.

## Results

### Study selection

An electronic search of the databases and references of relevant papers was conducted on 20-11-2022. 1220 studies were identified in total. The results of the search were stored in Endnote online (Clarivate Analytics, Philadelphia, USA), which identified 199 duplicate articles and removed them leaving 1021 remaining articles. Search strategies and the results of the searches for each database are listed in, Supplementary Appendix A, (Medline), Supplementary Appendix B, (Embase), Supplementary Appendix C, (Cochrane Database) and Supplementary Appendix D, (Web of Science), under supplementary data.

**Fig. 1:**
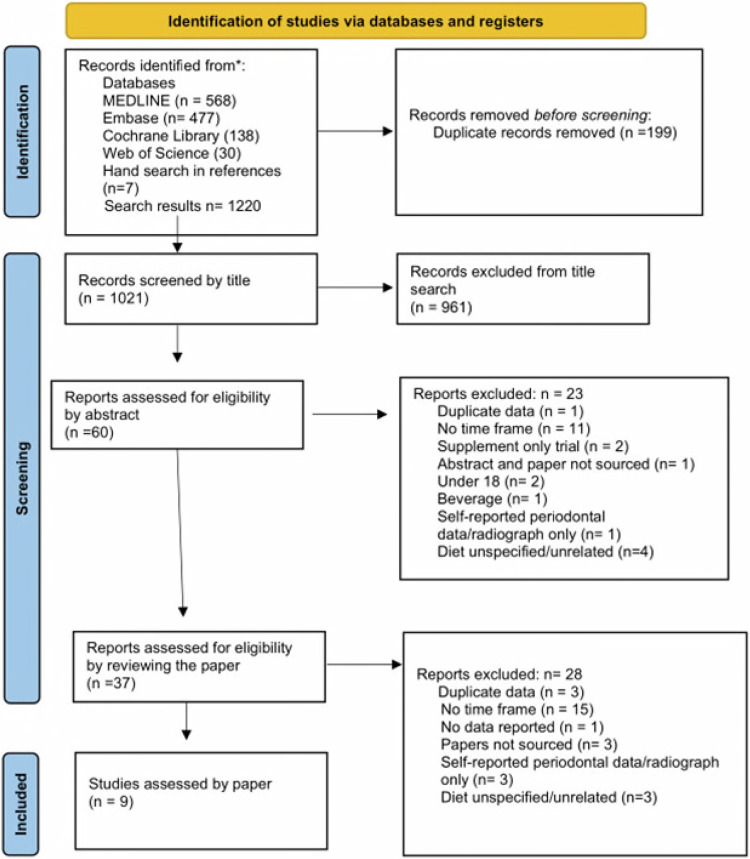
PRISMA flow diagram search strategy. Flow diagram (PRISMA- Preferred Reporting Items for Systematic review and Meta-Analyses) depicting the number of records identified, included and excluded and reasons for exclusion using the search strategy, inclusion and exclusion criteria for the systematic review.

A title search of the 1021 articles, excluded 961 articles, resulting in 60 papers for further review. Two independent examiners applied the exclusion/inclusion criteria to the abstracts of the 60 articles and excluded 23 of them, the reasons for exclusion were reported in the PRISMA Flow Chart in Fig. 1. 37 studies were identified for full paper searches; however, 3 papers were only available as abstracts, no further data was obtained thus these papers were excluded. 33 papers were reviewed in full; 24 studies did not meet the inclusion/exclusion criteria. Nine studies fulfilled the criteria, thus were eligible for inclusion in the systematic review which are listed in Table 4^64–72^. Supplementary Appendix E is the proforma used to apply the exclusion criteria including reasons for exclusion for each study, available in supplementary information.

**Table 4:** Summary of the statistical measurement and outcomes against the type of periodontal factors and diets that were monitored.

Study	Method of measurement	Results	Outcomes of periodontal indices measured, (Y = factor measured, significant reduction^a^, significant increase^b^)				Areas of diet investigated showing significance		Interpretation of results
			PPD	GI	CAL/ABL	BOP	Significance and GRADE	Diet specification and adherence	
Schwartz et al., USA, 2011	Multivariate hazard regression analysis, hazard ratios, (HR)	HR 0.95(CI = 95%, 0.91–0.99), HR 0.86 (CI = 95% 0.78–0.95) over 65 years	Y^a^		Y^a^		Yes, low	Increased proportions of fibre, fruit and vegetables (compliance scale)	PPD/ ABL, decreased in highest intake of fruit and vegetable group, very weak, 5%.
Yoshihara et al., Japan ^69^	Standard Coefficient, stepwise multiple regression analysis	−0.16 (*p* = 0.001), (*p* < 0.05)			Y^a^		Yes. low	Increased proportions of yellow and green veg, (compliance scale)	CAL reduced in highest intake of green and yellow vegetable group, weak correlation
Woelber et al., Germany ^70^	Mean values, standard deviations	GI = 0.03 (*p* < 0.05), inter group value	Y^b^	Y^a^		Y	Yes, moderate	Anti-inflammatory diet/western diet. Weight change and waist circumference confirmed adherence	GI decreased in intervention group, weak/moderate correlation, (PPD increased)
Bartha et al., Germany ^64^	Mean values, standard deviations and CI, (*p* = 0.05),	BOP = *p* = 0.045 (*p* < 0.05) inter group value	Y^b^	Y		Y^a^	Yes, moderate	Mediterranean diet/western diet. Weight change and waist circumference confirmed adherence	BOP reduced in intervention group, weak correlation.
Javid et al., UK ^67^	Not reported	No significance	Y		Y	Y	No, low	Antioxidant diet, increased fruit, veg and wholegrain, mean intakes compared with control- normal diet in UK.	No difference between groups
Eberhard et al., Australia, 2021	Mean differences and standard deviation, ANOVA (*p* = 0.05)	Semi-veg high/fat diet, (CAL = −5.11 +/− 9.68, *p* = 0.39), ANOVA, omnivorous versus semi vegetarian, (CAL, >1 mm *p* = 0.0266), (PPD > 1 mm *p* = 0.0138),	Y^a^		Y^a^	Y	Yes, moderate	Increased proportion of vegetables/high fat with alternative diets	CAL reduced in semi-veg high fat group, weak association. CAL and PPD reduced with semi vegetarian diet compared to omnivorous diet when factoring in confounding factors
Dodington et al., Canada^66^	ANCOVA	*P* = 0.026, (*P* < 0.05)	Y^a^			Y	Yes, low	Anti-inflammatory/antioxidant diet, increased proportions fruit and veg, dietary vit C, dietary B carotene, (compliance scale)	Only in non-smokers
Jauhiainen et al., Finland^71^	Incidence ratios (IR) with 95% confidence intervals (CI)	BSDS = 0.94 (95% CI 0.92,0.96) non-smokers 0.85 (95% CI 0.82–0.88), RFDS = 0.91 (95% CI −0.88, 0.94) non-smokers 0.74(95% CI 0.71–0.77)	Y^a^				Yes, low	Finish and Nordic diet, high veg, fish, (compliance scale).	Minimal decrease in PPD in whole group. Non-smokers group had an increased decrease in PPD compared with smokers.
De Angelis et al., Italy^65^	Coefficient, linear regression	FMBSD = −1.88, PPD = −0.34	Y^a^		Y	Y^a^	Yes, low	Optimized diet/non- optimised diet	PPD and FMBSD decreased in intervention group, (PPD weak/moderate), (BOP strong)
Outcomes		Ratios	PPD = 4^a^, 2 nil, 2^b^	GI = 1^a^, 1 nil	CAL = 2^a^ 2 nil	BOP = 2^a^ 4 nil	Grade	PPD- Low, GI high, CAL low, BOP moderate.	Mild improvement in 50% of periodontal parameters

*Y* Parameter was recorded, *CAL/ABL* clinical loss of attachment/alveolar bone loss, *PPD* periodontal pocket depth, *GI* gingival index, *BOP* bleeding on probing, *FMBSD* full mouth bleeding scores, *CI* confidence intervals, *IR* incidence ratios, *HR* hazard ratios.

^a^Periodontal parameter improved.

^b^Periodontal parameter worsened.

### Study characteristics

The characteristics of the nine eligible studies are summarised in, Supplementary Appendix F. Four studies were randomised control trials, one study was a clinical controlled trial and four were observational cohort studies.

The studies were published between 2011 and 2022 but the data ranged from 1984 through to 2022 and the duration of the studies ranged from 4 weeks to 24 years. The geographical locations were diverse but all of the participating countries were, ‘economically affluent’, in global terms^73,74^. Participant numbers varied from 30, to 625 and the age range spanned from 18 to over 75 years, however, the majority of subjects were in age groups of 50 and above. All but one trial, Schwartz et al., accounted for gender differences^68^.

The range of periodontal parameters measured between the studies included PPD, (Periodontal pocket Depth), GI, (Gingival Index) BOP, (Bleeding on Probing) and CAL/ABL, (Clinical attachment loss/Alveolar bone loss), seven of the nine studies measured multiple parameters. The summary of the parameters measured in each study are summarised in Table 4^64–68,70,72^.

Javid et al., omitted any data regarding periodontal indices^67^. The outcomes for the individual indices varied between studies, with 4 studies reporting improvements in periodontal pocket depth, 2 reporting improvements in clinical attachment, 2 reporting improvements in gingival indices and one reporting no changes in periodontal indices, (Table 4).

The studies utilised questionnaires and diet recall diaries to record and analyse the subjects’ dietary intake but their methods of recording and analysing the data differed between studies. All studies included diets associated with healthier diets compared to unhealthier diets. However, diet composition and analysis varied between studies and also between individual subjects.

The four RCT’s and the clinical control trial, relied on patients to adopt a healthier diet through educational support training, the remaining studies simply monitored the participants’ dietary habits with food frequency questionnaires. Eberhard et al., was the only study to provide the participants with the specified food^72^, (Supplementary Appendix F, Summary of Study Characteristics).

### Risk of bias across within and across the studies

RCT’s, non-RCT’s and cohort studies have different domains of bias. Therefore, the studies were divided into subgroups where the most appropriate risk of bias tool was applied according to the study design and the results represented as traffic light plots. A bespoke template was used to assess each study for risk of bias which was transferred to the Cochrane risk of bias tools. The template and results can be found in supplementary data labelled, Supplementary Appendix G.

**Fig. 2:**
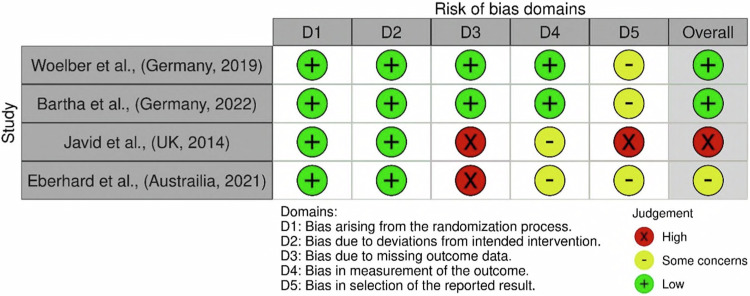
Risk of bias traffic light plot for RoB 2 (Risk of Bias) Cochrane risk of bias tool. The figure depicts the results from the identified and selected randomised control trials within the systematic review.

**Fig. 3:**
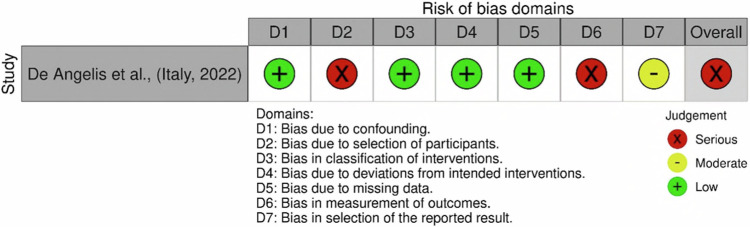
Risk of bias traffic light plot for ROBINS-I (Risk Of Bias In Non-Randomised Studies of Interventions) Cochrane risk of bias tool. The figure depicts the results from the identified and selected non-randomised control trial identified within the systematic review.

**Fig. 4:**
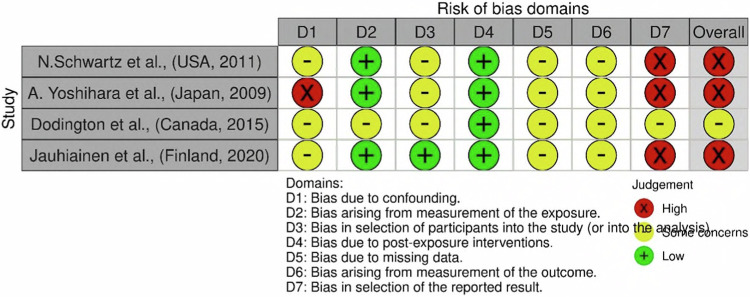
Risk of bias traffic light plot for ROBINS-E (Risk Of Bias In Non-Randomised Studies- of Exposure) Cochrane risk of bias tool. The figure depicts the results from the observational epidemiological studies identified within the systematic review.

The Cochrane Risk of Bias, RoB 2, assessment tool was applied to the four RCT’s, Woelber et al., Bartha et al., Javid et al., and Eberhard et al., all represented in Fig. 2^64,67,70,72^. The Cochrane ROBINS-I tool was applied to assess the non-randomised control trial by De Angelis et al., represented in Fig. 3^65^. Finally, the Cochrane ROBINS-E tool was used to assess the four remaining cohort studies, Schwartz, Yoshihara et al., Dodington et al., and Jauhiainen et al., represented in Fig. 4^66,68,69,71^.

### Risk of bias across the domains

Across the domain’s, the highest risk of bias was associated with reporting bias. Schwartz et al., found significant changes in periodontal indices in participants eating healthier foods when they divided the data set by age, with significant improvement in subjects over the age of 65 but not in those below 65, implying the groups may have been subdivided with the risk that this could represent data fishing^68^. Jauhiainen et al., found a stronger association between a healthier diet and non-smokers than that of smokers^71^. Dodington et al., only found significant differences amongst the non-smoking participants consuming a higher level of healthier food but found no change in the smoking group^66^. If the subgroup analysis in some of these studies had not been conducted, it is likely the studies would not have been reported as the overall effect would not have been significant.

Missing data lowered the quality of evidence further with high dropout rates for all of the cohort studies and for the Javid et al., study^67^. However, higher dropout rates are common for studies conducted over longer durations of time due to reasons such as migration, illness, difficulty complying with the study principles or death. The randomised control trials demonstrated a low risk of bias for randomisation but due to the nature of the study design, the participants could not be blinded to the intervention.

The cohort studies demonstrated low a risk of bias for measurement of outcomes as they were conducted with general surveys, thus the examiners would have been blinded to the authors intentions.

### Risk of bias within the studies

The quality of the individual studies ranged from moderate to low, with the RCT’s rating higher than the cohort studies, which would be expected.

Bartha et al., and Woelber et al., had low participant numbers which may have underpowered the study findings that an optimised diet significantly improved periodontal parameters; however, the participant numbers did meet the calculated power for the study designs, thus they were rated high quality of evidence^64,70^. The Eberhard et al, failed to conduct a power calculation, meaning the study may have failed to reach the appropriate effect size for a valid outcome, thus the study was rated, ‘moderate quality of evidence’, despite being and RCT^72^. Javid et al., failed to achieve the minimal number of participants required for the power calculation and failed to provide any data consequently lowering the quality of evidence^67^.

### Synthesis of results

A meta-analysis of the results was not plausible due to the high heterogeneity between the studies because of the different periodontal indices measured, the variation in diet and diet verification, the diversity of trial designs and duration, the diversity in subject age and the diversity in elimination or inclusion of confounding factors, therefore a narrative synthesis was used.

Eight of the studies reported a positive correlation between improvement in periodontal indices in the groups adhering to a healthier diet. However, none of the studies reported effective changes across all of the periodontal indices despite most studies recording multiple indices. Table 4 summarises the periodontal indices recorded in each study and the results of the individual studies.

### No changes in periodontal parameters

Javid et al., noted no difference in periodontal indices in patients adhering to a healthier diet^67^.

### Changes in PPD, in relation to an optimal diet across the studies

De Angelis et al., (Coefficient −0.34), Schwartz et al., (HR = 0.95 per serving (95% CI = 0.91–0.99)), Dodington et al., *P* = 0.026, (*P* < 0.05), Jauhiainen et al., BSDS, IR, (incidence ratios) 0.94 (95% CI 0.92, 0.96) non-smokers, 0.85 (95% CI 0.82–0.88), RFDS, 0.91 (95% CI −0.88, 0.94) non-smokers 0.74(95% CI 0.71–0.77), all demonstrated a mild/moderate reduction in PPD associated with the healthier diet options^65,66,68,71^. Eberhard et al., found a mild reduction in PPD (*p* = 0.0193) in the high fat group compared with the high carbohydrate group and in the semi vegetarian group compared with the omnivorous group, (*p* = 0.0138), where *p* = 0.05^72^.

However, improvements only occurred in participants aged over 65 for Schwartz et al. and principally in the non-smoking groups for Dodington et al., and Jauhiainen et al.^66,68,71^.

### Changes in CAL, in relation to an optimal diet

Eberhard et al., found a mild improvement in CAL < 5 mm MD, (mean difference) −5.11 +/− 9.68, (*p* = 0.039, *p* < 0.05), in the semi vegetarian/ high fat diet groups when compared with omnivorous high fat/high carbohydrate and semi vegetarian high carbohydrate diets^72^.

Yoshihara et al., demonstrated a minor association between a reduction in CAL and a high consumption of green vegetables, standard coefficient −0.16 (*p* = 0.001), (*p* < 0.05)^69^.

### Changes in BOP, in relation to an optimal diet

Woelber et al., (*P* = 0.45), De Angelis et al., (Coefficient −1.88), and Bartha et al., (*p* = 0.046), (*p* < 0.05), demonstrated a reduction in gingival bleeding, in the group of participants adhering to the healthier diets^64,65,70^.

### Changes in GI, in relation to an optimal diet

Woelber et al., (*p* = 0.03), and Bartha et al., (*p* = 0.178), demonstrated a significant reduction in GI in the intervention group where *p* < 0.05^64,70^. However, the control group also experienced a reduction in GI, (*p* = 0.178), in the Bartha et al., study^64^.

### Variables Influencing outcome

#### Confounding factors

All of the studies applied exclusion criteria or accounted for confounding factors known to influence periodontal health, however, these varied between the studies may have accounted for some of the variations in the results.

Woelber et al., and Bartha et al., applied the most thorough exclusion criteria, followed by Eberhard et al., Javid et al., and De Angelis et al., as would be expected from interventional studies^64,65,67,70,72^. The longitudinal cohort studies provided less information regarding exclusion criteria but all confounded factors associated with periodontal disease.

Dodington et al., and Jauhiainen et al., concurred that confounding for smoking influenced the reported outcomes of their studies, with little or no changes in periodontal indices in participants that smoked when eating an optimal diet compared with non-smokers^66,71^. Yoshihara et al., did not report on differences between smokers and non-smokers, confounding for smoking may have aligned Yoshihara’s findings more closely with that of Dodington et al., and Jauhiainen et al. ^66,69,71^.

Although many of the studies accounted for commonly known factors associated with periodontal disease such as smoking, diabetes and oral hygiene, new associations are continuing to be uncovered. Jauhiainen et al., eliminated participants with arthritis, whilst Eberhard et al eliminated participants with hyperthyroidism, however, evidence indicates that both conditions may impact periodontal health^71,72^. Schwartz et al. and Yoshihara et al., screened for, ‘healthy subjects’ but failed to disclose their definition of, ‘health’^68,69^. Health in an elderly adult may not be similar to ‘health’ in a young adult. None of the studies accounted for inherited genetic vulnerabilities, stress, sleep disturbance, nutritional deficiencies which are also factors suspected to influence periodontal health^2,22,75–79^. The failure to confound or exclude all of the factors that may influence periodontal disease could also account for the variance in results between studies.

## Discussion

The interventional studies applied similar exclusion and inclusion criteria, eliminated or accounted for confounding factors and applied similar principles to the definition of healthy and unhealthy foods. All of the studies except Javid et al., observed improved periodontal parameters in participants adhering to a healthy diet when compared with participants adhering to an unhealthy diet^67^.

Woelber et al., reported a 40% reduction in gingival bleeding, in patients adopting a, ‘low inflammatory diet,’ which Woelber claims to be comparable to adding interdental cleaning to a patient’s oral hygiene regime^70^. Bartha et al., reported an 11% reduction in BOP, a finding similar to the improvement in oral health in subjects using electric toothbrushes compared to manual toothbrushes over a three month period^64^. Both accounted for differences in oral hygiene by suspending all interdental cleaning during the trial and accounted for baseline dietary differences by selecting participants practicing a ‘Western Diet’. They also verified compliance to the specified diets by monitoring the participants weight changes, however, the numbers of participants were low.

Eberhard et al., reported a reduction in CAL and to a lesser extent, PPD, in participants adopting a semi-vegetarian diet, but failed to calculate the minimum effect size for the study and was rated low quality of evidence^72^. The control trial by De Angelis et al., also reported a large decrease in full mouth bleeding scores and a significant reduction in PPD, in the group adopting a healthier diet but was also rated, ‘low quality of evidence’, based on the customised risk assessment tool, Supplementary Appendix G^65^.

The cohort studies were only able to infer an association between participants eating a healthier diet and periodontal health rather than establishing a direct cause and effect between parameters measured. Nevertheless, the observational studies provided valuable insight regarding confounding factors that the randomised control trials omitted. Doddington et al., and Jauhiainen et al., inferred that, non-smoking participants eating a healthy diet were associated with improved periodontal parameters when compared with smokers^66,71^. A finding that would not have been evident within the studies that excluded smokers. Additionally, the cohort studies were conducted over long time spans and assessed larger numbers of participants compared with the interventional studies, with the exception on Javid et al. ^67^.

Javid et al. was the only study that did not report significant improvement in periodontal parameters in participants adopting a healthier diet^67^. Several factors may have contributed to the differing outcome. Primarily, Javid et al., did not exclude smokers from the study, nor did the author confound for differences between smokers and non-smokers, which may have influenced the outcomes, when factoring in the findings of Dodington et al., and Jauhiainen et al. ^66,67,71^. Secondly, it is possible that the participants regressed back to their original dietary habits over the extended time frame of six months which may be less of a risk in studies with a shorter time span. The extended trial time may also have accounted for the higher dropout rate resulting in participant numbers falling below the power of effect.

The combined evidence suggests that adopting a diet associated with improved outcomes for chronic systemic diseases with an inflammatory component, such as heart disease, obesity or diabetes, may positively impact periodontal parameters in patients with gingivitis or periodontitis, particularly in the older population but the effects may be negated by confounding factors such as smoking. However, the strength of the associations differed between studies as did, the periodontal parameters, the specified diets and the methods of measuring the diets which brings into question the validity of the findings.

A cross-sectional study by Li et al., observed that participants on a pro-inflammatory diet were 53% more likely to have periodontitis, compared with participants on a low inflammatory diet with stronger associations amongst older males^80^. A further six cross sectional studies investigating periodontal parameters with heathy eating, also, agreed with the findings of this review^81–86^. They were all excluded from the current review because they did not measure changes in diet and periodontal indices with time. Although cross-sectional observational studies are a weak evidence base, the corroboration between the findings combined with the larger populations and the wide geographical range associated with these studies strongly supports the findings of this review.

A systematic review by Saenz-Ravello et al., also concluded that a healthy diet may mildly positively influence periodontal parameters but advised the results should be interpreted with caution due to the diversity between studies^87^. Saenz-Ravello et al., differed from this study as they included elimination diets which this review omitted^55–57,79,88,89^. Skoczek-Rubinska et al., conducted a systematic review comparing intake of fruit and vegetables on periodontal disease and came to similar a similar conclusion but differed from this review which excludes studies on single foods^90^.

In contrast, Jeong et al., found no statistical relationship between periodontal parameters and a western diet but only reviewed two studies, neither of which met the inclusion/exclusion criteria for the current review^91^. Kulkarni et al., conducted a systematic review assessing the effect of nutrition on periodontal disease, focusing on supplements rather than whole foods but found the current evidence too limiting to determine a relationship, which may have been due to the lack of fibre within the supplements^46,58–61,92^.

### Limitations

Woelber et al., Bartha et al., and Eberhard et al., were conducted over a four-week period, which was adequate for monitoring a reduction in gingivitis but less appropriate for monitoring changes in periodontitis which progresses more slowly^64,70,72,93^. The shorter time frame of these studies may account for the differing outcomes for PPD and CAL when compared with the longer cohort studies.

Increasing the participant numbers in the interventional studies, would have strengthened the validity of the outcomes as lower participant numbers increase the risk of outliers influencing the outcomes^94^. De Angelis et al., did not apply random allocation to the groups and made no reference to blinding of the examiners throughout the study which brings into question the validity of the results^65^.

Eberhard et al., De Angelis et al., and Javid et al., failed to report on the initial dietary status of the study participants^65,67,72^. Thus, variation between subjects’ initial dietary habits may have influenced the impact that the intervention and control diets had on changes to periodontal parameters.

Eberhard et al., delivered the recommended diet to the participants but the remaining interventional studies left the participants to source their own food, which increased the risk of deviation from the recommended diet^72^. The method of diet validation was via self-reported diaries, which introduced the of risk recall inaccuracies or false reporting. The interventional groups were required to alter their diet to a healthier option, whilst the control group retained their old dietary habits. It is more of a risk that the intervention group reverted to their previous diet than the control group deviated from their current diet. If the null hypothesis was rejected, then failure to self-report non-compliance within the healthier diet group, would have resulted in less differences between control and interventional groups. Consequently, creating a type II error in favour of the null hypothesis, thus, improved accuracy in reporting of diet adherence would theoretically improve periodontal indices with more accurate reporting.

Similarly, the observational studies utilised self-reported food frequency questionnaires. In particular, Dodington et al., required participants to recall their dietary habits over the span of an entire year, which was unlikely to be an accurate^66^. Jauhiainen et al., only measured dietary exposure at the start of the eleven-year study, thus any changes in dietary habits over the time of the trial would not have been accounted for when comparing periodontal indices with time^71^. A video log may have been a more effective method to monitor diet adherence and is now practical with current technology.

Although there was a general consensus between studies regarding healthy diets and foods, variations in the types of questionnaires, variations in the estimated amounts of food and variations in the categorisation of food groups impeded comparisons. The observational studies used differing food frequency questionnaires, differing categories of healthy foods and differencing methods to quantify the comparison of unhealthy and healthy food groups.

### Conclusion and clinical relevance

The evidence reviewed within this systematic review implies that adopting a diet associated with improved outcomes for chronic systemic diseases with an inflammatory component, such as heart disease, obesity or diabetes, has the potential to improve periodontal parameters in patients with gingivitis or periodontitis, particularly in the older population but the effects may be negated by confounding factors such as smoking. However, the evidence was of low to moderate quality, the components of the diets and the methods used to record and analyse the diets were diverse and the confounding factors between studies were inconsistent, thus, the conclusions of this review should be interpreted with caution. Nevertheless, the findings indicate that further research may provide valuable insight regarding dietary habits and periodontal disease. A study of periodontal parameters conducted over a prolonged period of time, 10 years, with thousands of participants across a range of settings and locations, using a video monitoring system to record dietary habits may give further insight into the current findings. The evidence may then be used to train dental healthcare professionals regarding healthier eating options which could be incorporated into patient treatment plans in combination with oral hygiene instruction and non-surgical periodontal therapy, consequently optimising periodontal outcomes for patients.

Communities with a tendency to live longer with less health issues, associated with chronic inflammation, appear to consume a greater variety of plant matter and have a more diverse gut biome compared with communities associated with an increased tendency to systemic inflammatory conditions^95,96^. Understanding the mechanisms of how foods influence our health is a developing field of research and evidence suggests that diet not only influences nutritional imbalance but may also influence the host immune system, possibly via the host-gut microbiome which may subsequently influence systemic inflammation. Thus, research into the inflammatory potential of food and the host microbiome response may provide more insight into the role diet plays in systemic inflammatory conditions^61,96–100^.

## Supplementary information

Supplementary Information

Prisma checklist
