# Supplementary material for: Does adopting a healthy diet improve periodontal parameters in patients susceptible to periodontal disease? A systematic review
Source: Evid Based Dent. 2025 Jan 10;26(2):111. doi: 10.1038/s41432-024-01098-0 (PMC12204859; doi:10.1038/s41432-024-01098-0)
Supplement: Supplementary file 1 — Supplementary Information [file 41432_2024_1098_MOESM1_ESM.pdf]

Appendix A: Medline Search Results

Medline Search Strategy		
ID	Search term	Hits
1	exp *"diet, food, and nutrition"/ or exp *diet/	1176706
2	periodontitis.mp or exp * Chronic Periodontitis/ or exp * Aggressive Periodontitis/ or exp *Periodontitis/	37801
3	Limit 2 to humans	32970
4	1 and 2 and 3	568

Appendix B: Embase Search Results

Embase (Medline) Search Strategy		
ID	Search term	Hits
1	exp *"diet, food, and nutrition"/ or exp *diet/	984793
2	periodontitis.mp or exp * Chronic Periodontitis/ or exp * Aggressive Periodontitis/ or exp *Periodontitis/	49254
3	Limit 2 to humans	42084
4	1 and 2 and 3	477

Appendix C: Cochrane Search Results

Cochrane Search Date run 29/09/2022		
ID	Search Term	Hits
#1	[mh diet]	20442
#2	[mh food]	38399
#3	[mh gingivitis]	1521
#4	[mh periodontitis]	3223
#5	#1 or #2	50111
#6	#3 or #4	4563
#7	#5 and #6	138

Appendix D: Web of Science Search Results

Web of Science Search Strategy		
ID	Search Term	Hits
1	(((((ALL=(diet)) OR ALL=(food)) AND ALL=(periodontitis)) OR ALL=(gingivitis)))	9207
2	ALL=(diet)	615554
3	ALL=(food)	2056610
4	ALL=(periodontitis)	34845
5	ALL=(gingivitis)	8274
6	ALL=(human trials)	392174
7	#2 OR #3	2473856
8	#4 OR #5	40017
9	#7 AND #8	1307
10	#9 AND #6	30

Appendix E: Proforma with reasons for inclusion and exclusion applied to abstract and full paper search

Author	Year	Study	Abstract-Reason for rejection after Inclusion/exclusion criteria applied	Full paper- Reason for rejection after inclusion/exclusion criteria applied	Accepted papers
Abbass, M	2019	The occurrence of periodontal disease and its correlation with different risk factors among a convenient sample of adult Egyptian population: cross sectional study		No time frame	N
Adegboye, A	2016	Calcium, vitamin D, casein and whey protein intakes and periodontitis among Danish adults	Supplement	/	N
Al-Zahrani, M	2005	Periodontitis and three health enhancing behaviours; maintaining normal wieght, engaging in recommended level of exercise and consuming a high level of diet	No time frame	/	N
Alhassani, A	2020	Dietary intake and risk of periodontitis		Self-reported periodontal data	N
Alsyefi, A	2020	Dietary habits and periodontal health in Saudi Arabia: A qualitative study		No data	N
Altun, E	2021	Association between dietary pattern and periodontitis-a cross-sectional study		No time frame	N
Atarbashi-Moghadam, F	2020	Effects of raw vegan diet on periodontal and dental parameters		No time frame	N
Barbosa, M	2021	Assessing the Association Between Nutritional Status, Caries, and Gingivitis in Schoolchildren: A Cross-Sectional Study	Under 18	/	N
Bartha, V	2021	Suitablility of three dietary assessment tools in the background of dental medicine	No topic related	/	N
Bartha, V	2022	Changes in serum omega fatty acids on a Mediterranean diet intervention in patients with gingivitis: An exploratory study		Duplicate data	N
Bartha V	2022	Effect of the Mediterranean diet on gingivitis: A randomized controlled trial			Y
Bawadi H	2011	The association between periodontal disease, physical activity and healthy diet among adults in Jordan		No time frame	N
Bidinotto A	2022	Investigation of direct and indirect association of ultra-processed food intake and periodontitis		No time frame	N
Bilgnaut	1992	High Fruit consumption and periodontal status in farm workers		No time frame	N
Botelho	2021	Periodontal Health, nutrition and anthropometry in professional footballers: A preliminary study		No difference in diet between candidates	N
Botelho	2021	The Role of Inflammatory Diet and Vitamin D on the Link between Periodontitis and Cognitive Function: A Mediation Analysis in Older Adults		No time frame	N
Campan P	1997	Pilot study on n-3 polyunsaturated fatty acids in the treatment of human experimental gingivitis	Supplements	/	N
Cassiano L	2022	Periodontitis Is Associated with Consumption of Processed and Ultra-Processed Foods: Findings from a Population-Based Study		No time frame	N
Costa P. D	2021	Influence of micronutrient intake, sociodemographic, and behavioural factors on periodontal status of adults assisted by a public health care system in Brazil: A cross-sectional multivariate analysis	No time frame		N
Costa P. D	2022	Investigating oral and systemic pathways between unhealthy and healthy dietary patterns to periodontitis in adolescents: A population-based study		No time frame	N
De Angelis P	2022	The Effect of an Optimized Diet as an Adjunct to Non-Surgical Periodontal Therapy in Subjects with Periodontitis: A Prospective Study			Y
Doddington D	2015	Higher intakes of fruits and vegetables, beta-carotene, vitamin C, alpha-tocopherol, EPA, and DHA are positively associated with periodontal healing after nonsurgical periodontal therapy in nonsmokers but not in smokers			Y
Eberhard J	2022	A randomized clinical trial to investigate the effect of dietary protein sources on periodontal health			Y

Gaengler P	1986	The effects of carbohydrate-reduced diet on development of gingivitis		No paper found	N
Gronwald B	2022	Influence of Freeze-Dried Diet on Oral Hygiene Indicators in Strict Isolation Condition of an Analog Space Mission		No diet comparison	N
Iwasaki M	2013	Dietary antioxidants and periodontal disease in community-based older Japanese: a 2-year follow-up study		Duplicate data	N
Jauhianinen L	2018	Periodontal condition in relation to the adherence to nutrient recommendations in daily smokers		Duplicate data/no time frame	N
Jauhianinen L	2020	Poor diet predicts periodontal disease development in 11-year follow-up study			Y
Klok S	2019	Salivary IL-1s but not dietary flavonoids are associated with healing after periodontal therapy to prevent tooth loss	Beverage		N
Kwon Y	2022	How Much Does Plant Food (Fruits and Vegetables) Intake Affect the Prevention of Periodontal Disease in the Korean Elderly?		No time frame	N
Lee J	2017	The association of dietary Vitamin C intake with periodontitis among Korean adults: Results from KNHANES IV	No time frame		N
Lee J	2022	The association of food insecurity with tooth decay and periodontitis among middle-aged and older adults in the United States and Korea		No time frame	N
Li A	2021	Dietary inflammatory potential is associated with poor periodontal health: A population-based study		No time frame	N
Li W	2022	The association between dietary vitamin C intake and periodontitis: result from the NHANES (2009-2014)	No time frame		N
Machado V	2021	Association between dietary inflammatory index and periodontitis: A cross-sectional and mediation analysis		No time frame, duplicate data	N
Mapare S	2013	A comparative clinical evaluation of diet intake and effect of various nutrition on aggressive periodontitis patients		Periodontal diagnosis on radiograph only	N
Marruganti C	2022	Association between dietary inflammatory index and periodontitis: A cross-sectional and mediation analysis		No time frame	N
Maruyama S	2011	Relationship between dietary intake and the clinical parameter of periodontitis in perimenopausal Japanese female patients		Paper not found	N
Maruyama S	2013	Relationship between nutrition intake and periodontal condition in female japanese periodontitis patients		Paper not found	N
Merchant	2006	Whole-grain and fibre intakes and periodontitis risk in men	No periodontal exam conducted as part of the study, report from a dentist and radiographs only		N
Milledge K	2017	Dietary intake of nutrients and compromised periodontal health: The concord health and ageing men project		No measurement of periodontal indices	N
Nascimento G	2017	Diet-Induced Overweight and Obesity and Periodontitis Risk: An Application of the Parametric G-Formula in the 1982 Pelotas Birth Cohort	Diet not specified; diet induced obesity		N
Nielsen S	2016	Dietary fibre intake is inversely associated with periodontal disease among US adults	No time frame		N
Oreskovic J	2022	Hypertensive renal transplant recipients adhering to Mediterranean diet present better periodontal status	No time frame		N
Ottosson F	2022	The inverse association between a fish consumption biomarker and gingival inflammation and periodontitis: A population-based study	No time frame		N
Park H	2015	Change of periodontal inflammatory indicators through a 4-week weight control intervention including caloric restriction and exercise training in young Koreans: a pilot study	Diet not specified; diet induced obesity		N

Petrenya N	2022	Dietary patterns association with periodontitis and number of teeth. Data from the Tromso study: Tromso 7	Paper not found		N
Petti S	2000	Nutritional variables related to gingival health in adolescent girls	Under 18		N
Radic J	2022	Interconnectedness between periodontitis stage, oral hygiene habits, adherence to the Mediterranean diet and nutritional status in Dalmatian kidney transplant recipients: a cross-sectional study		No time frame	N
Salazar C	2018	Better-quality diet is associated with lower odds of severe periodontitis in US Hispanics/Latinos	No time frame		N
Schwartz N	2012	High-fibre foods reduce periodontal disease progression in men aged 65 and older: the Veterans Affairs normative aging study/Dental Longitudinal Study			Y
Staudte H	2012	Comparison of nutrient intake between patients with periodontitis and healthy subjects	No time frame		N
Terashima T	2017	The association between dental health and nutritional status in chronic obstructive pulmonary disease	Diet not specified		N
Tomofuji T	2011	Relationships between eating habits and periodontal condition in university students		No time frame	N
Ward W	2015	Higher intakes of fruits and vegetables, alpha linolenic acid, vitamin E and beta carotene are associated with improved periodontal healing after periodontal therapy	Duplicate data/Doddington		N
Woelber J	2019	The influence of an anti-inflammatory diet on gingivitis. A randomized controlled trial			Y
Woelber J	2021	Effects of a non-energy-restricted ketogenic diet on clinical oral parameters. An exploratory pilot trial		Exclusion diet	N
Wright D	2020	Association between diet and periodontitis: A cross-sectional study of 10,000 NHANES participants	No time frame		N
Yoshihara A	2009	A longitudinal study of the relationship between diet intake and dental caries and periodontal disease in elderly Japanese subjects			Y
Zare A	2014	Impact of a customised dietary intervention on antioxidant status, dietary intakes and periodontal indices in patients with adult periodontitis			Y

Appendix F: Summary of Characteristics of Studies

Study Data	Study Design	Time	Number of subjects	Age	Inclusion Criteria	Exclusion Criteria	Confounders	Randomisation	Blinding	Initial Periodontal Status	Initial Dietary status	Intervention/ Group of Interest	Control/ comparator	Power	Periodontal Parameter	Diet record	Statistical data and significance	Outcomes Reported
Bartha *et. al.*, Germany 2022 University Tubingen	Single blind RCT	6 weeks	37, (17 males,20 females)	18-49	Western diet, (Cena and Calder, 2020)	Periodontitis, smoking, pregnancy, recent antibiotic history, anti-inflammatory drugs, medication affecting BOP, probiotics, food intolerance, eating disorder, plant-based diet	Age, sex,	Yes	Dentist	Gingivitis	Western diet	Mediterranean Diet	Western diet	Yes	BOP, GI, PPD	2 MD training sessions initially, 2 more 60 minutes at week 2 before week 4 and week 5., T1 and T2, DEGS-FFQ and MEDAS diet screener	BOP decreased P = 0.045 inter group.	BOP decreased
Eberhard *et. al.*, Australia, 2021	Double blind RCT	4 weeks	67	66-75	Health, MBI 20-35kg/m sq.	Diabetes mellitus, renal/levier disease, cancer, hyperthyroidism, weight medication, weight loss>10% over 5 years, current smokers, >3 alcoholic drinks per day, vegetarians, food intolerances	Sex, BMI, smoking	Yes	Participants	Not stated	Not specified	(semi-vegetarian low carb/high fat) :(semi veg high carb/low fat)	(Omnivore high fat/low carb) :(omnivore high carb/low fat)	No	PPD BOP CAL	7-day FFQ at baseline and week 4	Semi-veg high/fat diet, compared with alternative groups (CAL= -5.11 +/- 9.68, p=0.39). ANOVA, omnivorous versus semi vegetarian, (CAL, p= 0.266), (PPD, p=0.138)	Semi vegetarian high fat diet group had significantly decreased, CAL. Semi vegetarian groups had significant reduction in PPD and CAL compared with omnivorous groups
Javid *et. al.*, UK, 2014, Hospital	Single blind RCT	6 months	37 (male=female ratio)	30-65	30-65 years, BMI > 18.5kg m sq., periodontitis	Type 2 diabetes, BMI< 18.5kg m sq., > 20 cigarettes per day, drug/alcohol abuse, pregnancy, weight change > 2kg in last month, >500g fruit per day	Age sex, BMI, smoking.	Yes	Researcher collecting outcomes was blind to participants	Chronic periodontitis.	Consuming less than 500g fruit and vegetables per day	Whole grain, fruit and veg intake, individual diet plan	No diet intervention or advice	Not met, n=44	BOP PPD CAL	Food and lifestyle questionnaire. One to one diet package and training. 3-day food diary T0, T1 (3 months), T3 (6 months)	No data	No difference

Woelber *et. al.*, Germany 2019, Frieberg University	Single blind RCT	6 weeks	30, (13 males, 17 females)	18 +	Western diet, processed carbohydrate intake above 45%	Periodontitis, smoking, severe illness, pregnancy, recent antibiotic history, anti-inflammatory drugs, medication affecting BOP, diabetes	Age, sex, PI, Vit C, Vit. D, BMI omega 3 oils	Yes	Dentist	Gingivitis,	Western Diet	Optimised diet	Western diet	Yes	BOP GI PPD	Instruction in diet, 24 diet recall diary for 1 week at week 2,5 and 8. PRODI software analysis	Baseline GI 1.04+- 0.21, End GI 0.61+-0.29, (p <0.05), mean with standard deviation	GI significantly reduced in optimised diet group
De Angelis *et. al.*, Italy, 2022	Prospective Clinical Trial	6 months	60, (27 males, 33 females)	18+	Adult with chronic periodontitis	Uncontrolled systemic disease, radiation therapy, pregnancy, drugs influencing gingival health, physical impairment to OHI, smoker, recent, recent periodontal therapy, recent AB history	Age gender weight BMI height obesity	No	None	Periodontitis	Not specified	Optimised diet, (OD)	Non-optimised diet (ND) n=32	Yes	PPD, CAL, GI	3-day food diary, analysed by computer software, taken just after intervention and at the end of the trial, 4-week diet program provided for OD participants, week 4 further diet advice,	PPD -0.32 coefficient, FMBSD -1.88, linear regression model	PPD and BOP decreased in intervention group, (PPD weak/moderate), (BOP strong
Dodington *et. al.*, Canada, 2015	Prospective observational cohort	4 months	86, (43 males, 43 females)	18+	Chronic periodontitis	Not stated	Smoking BMI age gender diabetes	No	Not stated	Chronic periodontitis	Variable, individually assessed at baseline	Observational III tertial-highest intake fruit and veg with periodontal status after scale	Observational I tercile, lowest intake of fruit and veg with periodontal status after scale	No	BOP, PPD	2005 BLOCK FFQ (110 food items) reviews diet over the previous year	PD P= 0.026, smokers only	Significant reduction in PPD in non-smokers but not smokers for fruit, veg, b-carotene, vitamin C

Jauhiainen *et. al.*, Finland 2020	Longitudinal cohort	11 years	240, (84 males, 156 females)	30+	No chronic periodontal disease,	Diabetes, rheumatoid arthritis, >50 years,	Smokers/non-smokers, education/nsaids/dental attendance/medications/exercise	No	Not stated	Healthy	Not specified	Fruit, veg, berries, fish, oats, barley, low fat milk, fat PUFA, white meat	Low quartiles 0,1, (red meat, total fat, alcohol, salt sucrose)	No	PPD	FFQ over previous year (128 food items), BSDS (Baltic Sea diet score) RFDS (Recommended Finish Diet Score), amalgamated into a 0-3 quartile scoring system, low being unhealthy	BSDS= 0.94 (95% Cis 0.92,0.96) non-smokers 0.85 (95% Cis 0.82-0.88), RFDS= 0.91 (95% Cis -.88, 0.94) non-smokers 0.74(95% Cis 0.71-0.77)	Minimal decrease in PPD in whole group. Non-smokers group had an increased decrease in PPD compared with smokers.
Schwartz *et. al.*, USA, 2011, USA, Boston	Longitudinal cohort	1984-2009 average 15 years range 2-24 years	625 males	18+	Medically healthy	No teeth, no follow up appointments after 1984, drop outs	Age smoking frequency of brushing interdental cleaning BMI education exercise	No	Not stated	Not stated	Not specified	Highest intake of fruit and veg as source of fibre with time	No control, comparator lowest intake of fruit and veg as source of fibre	No	PPD	FFQ at each visit, 126 food items	0.95(CI =95%, 0.91-0.99), over 65 years	High intake fibre from fruit and veg is associated with decreased PPD in over 65 men
Yoshihara *et. al.*, Japan, 2009	Longitudinal cohort	6 years	261, (144 males, 117 females)	70	Good health, no special care needs	Not stated	BMI Gender Education	Yes	Not stated	Not stated	Not specified	Dark green and yellow veg, (other vegetables and fruits), (fish, shellfish, meat, beans and eggs)	No control, comparator (Cereals, nuts, seeds, sugar and sweeteners, confectioneries), (Fats, oils)	No	CAL	Semi-quantitative FFQ, baseline interview, 6 food groups, conducted annually, 7 times in total	- 0.16 (p=0.041),	Increased intake of dark green and yellow vegetables is associated with reduction in CAL

RCT BMI=Body mass index, PPD= periodontal pocket depth, CAL=clinical attachment loss, BOP=bleeding on probing, GI= gingival index, FMBS= full mouth bleeding scores, CI= confidence Intervals, FFQ= food frequency questionnaire, BSDS= Baltic sea diet score, RFDS recommended Finnish diet score, OD=optimised diet, ND- non-optimised diet, MD= Mediterranean diet, MEDAS= Mediterranean Diet Adherence Score, ANOVA= Analysis of variance, PUVA=polyunsaturated fats, DEGS=German National Health Survey for Adults.

Appendix G: Table of Risk of Bias Assessment tool

Study		Selection Bias				Performance Bias					Detection Bias		Attrition Bias	Reporting Bias	overall
	Participant selection, sample representativeness	Sequence generation	Allocation concealment	Confounding factors at baseline accounted for, age sex, socioeconomics	Confounding factors included in comparison or excluded	Blinding of personnel	Blinding of participants	Data by specialists, non-specialists, self-reported	Power calculation	Classification of interventions/ factors being assessed	Blinding of Outcome Assessment	Deviations from intended intervention	Missing data		
N.Schwartz *et al*. USA, 2011	Boston area, males, **medium**	Observational, no prospective group allocation	Not applicable, examiners taking data for a survey	Only males but similar region and social mobility- medium	Minimal reference to confounding factors- **medically healthy** medium	Single periodontist, unaware of the diet assessment, data collection not conducted by the personnel analysing the data- **low**	Yes	Single calibrated perio specialist, self-reported FFQ	No	upper and lower ranges- medium	Examiners and authors were independent, but examiners may have chosen the trial data because the evidence was supportive- medium	No, low	no, low	Yes, authors split results into over 65 and below 65, high	High
A. Yoshihara *et al.* Japan, 2009	Japanese elderly Nigata, randomly selected from those responding to mailed questionnaires, randomisation method not stated, University dental department- medium	Observational, no prospective group allocation	Not from dietitian, dentist unclear- Medium	Yes- medium	No-only mentions good health requiring no special care -high	No but separate dietitian and periodontist, unclear if they had access to the trial data, 4 periodontists were used and calibrated- medium	Yes	4 calibrated denturists assessed periodontal health; dietitians assessed food intake	no-high	upper and lower ranges- medium	Examiners were independent but unclear if they were blinded from the outcomes or group allocations.	No, low	no, low	Yes, authors were aware of objectives when interpreting data, high	High
Woelber *et al.* Germany 2019	Within Dental hospital, patients were paid -high	Recruitment not randomised, but randomisation into groups by computer software	Only dental examiners	Yes-low	Yes-low	Dental examiner blinded-low	Intervention was a diet change, participant not blinded- medium	Specialists recording periodontal parameters. Specialist diet advice, self-reported questionnaires- medium	yes-low	Yes-low	Yes- dentists blinded-low	No, low	No-low	Small sample size, so Medium but large effect so downgraded to low	Low

Bartha *et al.* Germany 2022	University, advertisements-medium	Recruitment not randomised, but randomisation into groups by computer software	Only dental examiners	Yes-low	Yes-low	Dental examiner blinded-low	Intervention was a diet change, participant not blinded-medium	Specialists recording periodontal parameters. Specialist diet advice, self-reported questionnaires-medium	yes-low	Yes-low	Yes- dentists blinded-low	No-low	No-low	Small sample size, so Medium	Low
Javid *et al.* UK, 2014	Hospital setting	Recruitment not randomised, but randomisation into groups by computer software	Not stated	Yes, no difference between groups at base line-low	Yes-but not all factors-medium	Not stated-high	No-medium	Specialists recording periodontal parameters. Specialist diet advice, self-reported questionnaires-medium-medium	Yes, but not met-High	Yes-low	Not stated, high	No-low	Yes-high	Researcher collecting outcome measures blind to group identity of patient-low	High
Eberhard *et al.* Australia, 2021	Nutrition fir healthy living study	Recruitment not randomised, but randomisation into groups by computer software-medium	Not Stated	No difference between groups-Low	Low	Unclear medium	Yes low	Specialists recording periodontal parameters. Specialist diet advice, with meals provided self-reported questionnaires-low	No calculation high	Yes-low	Not stated-high	No-low	No- low	Medium, Uses results from an RCT	Medium
Dodington *et al.* Canada, 2015	Periodontal clinic-2013-2014	Not randomised, all patients with PD 4mm or more were included-medium	Medium	Pt characteristics were accounted for Under reporting was accounted for; medical conditions were not accounted for medium	Yes smoking	Not stated-high	Not stated but observational, no intervention medium	Data specialists for hygiene and calibrated, self-questionnaire, computer analysed medium	Stated it was met but not specified-medium	Percentile comparison for diet, baseline and follow up data for PPD and BOP, low	Unclear	Not enough smokers in the study to compare smoking to non-smoking	no	Medium	Medium
Jauhiainen *et al.* Finland 2020	Health survey 2000 and 2011, THL Finish Institute for Health and Wealth	Observational, no prospective group allocation	Low	Yes medium	Yes-medium	Yes, personnel conducting data collection blinded low	Yes- low	Dental specialist, calibrated, self-questionnaire over 12-month recall, 2000 scoring system used- medium	no power calculation-high	upper and lower ranges-medium	Not to the person extracting the data, but the examiners carrying out the protocol were medium	Unable to determine if smokers benefit from diet more than nonsmokers,	No	Yes, authors were aware of objectives when interpreting data, high	High

												low numbers medium			
De Angelis *et al.* Italy, 2022	Dental clinic, private march 2020-october 2020	non randomised selection, pts unwilling to opt for diet plan were in the ND group-medium	No-high	Yes-low	Yes -low	no -high	No-high	specialist for perio. Specialist diet advice with food questionnaires-medium	yes -low	Yes-low	no-high	no-low	no-low	Reporting medium. Outcomes high	High
